# Supplementary material for: Public expectations about the impact of COVID-19 on climate action by citizens and government
Source: PLoS One. 2022 Jun 9;17(6):e0266979. doi: 10.1371/journal.pone.0266979 (PMC9182260; doi:10.1371/journal.pone.0266979)
Supplement: S1 File — (DOCX) [file pone.0266979.s001.docx]

**Supporting information**

**S1 Fig. Evolution of contagion and death cases in Spain (Confinement: 14 March - 21 June; survey: 23 June - 3 July)**

**
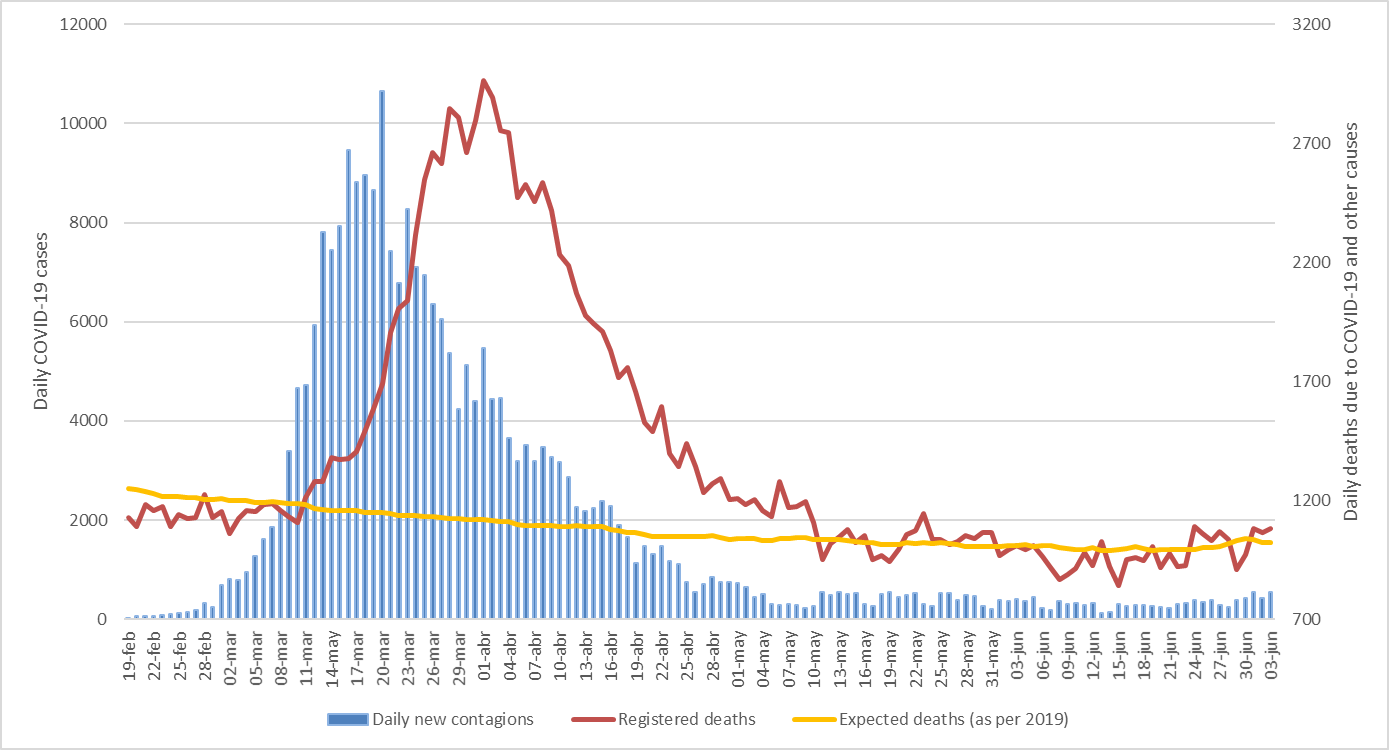
**

Source: Spanish National Epidemiology Center <https://cnecovid.isciii.es/covid19/#documentaci%C3%B3n-y-datos> (contagion cases), and Europa Press, [www.epdata.es](http://www.epdata.es) (death cases)

Survey questions

What is your education?

What is your postal code?

Nationality

What are in your view the main problems that exist in Spain? Take some time to read all the options and mark all that apply

- Poverty
- Water scarcity
- Climate change
- Terrorism
- The economic situation
- Armed conflicts
- Global population growth
- Proliferation of nuclear weapons
- Spread of infectious diseases
- Unemployment
- Corruption
- International migration
- Gender discrimination and violence
- Biodiversity loss
- Air pollution
- Other
- None
- other, which ones? _______
- I do not know

Two issues that receive a lot of attention nowadays are climate change and the Covid-19 (Coronavirus) pandemic. Overall, how do you think the Covid-19 pandemic will affect actions of the government towards climate change? (Response options: "Very negatively, negatively, neutrally, positively, very positively”)

Can you explain in your own words why you think that Covid Will affect positively/negatively the actions of the Spanish government with regard to climate change? We would like that you take your time to answer this question and write some sentences. All kinds of answers are welcome. [open response field] (Min 4 characters, no maximum)

Control question 1

¿Where do you live? (Response options: In an urban area, in a periurban area, in a rural area)

Overall, how do you think the Covid-19 pandemic will affect actions of the Spanish citizens towards climate change? (Response options: Very negatively, negatively, neutrally, positively, very positively)

Can you explain in your own words why you think that Covid Will affect (…) the actions of the Spanish citizens with regard to climate change? We would like that you take your time to answer this question and write some sentences. All kinds of answers are welcome. [open response field] (Min 4 characters, no maximum)

In this survey, exceptionally, you will participate in a draw for 100 Euros. The probability of winning is 1%, that is, 1 out of 100 collaborators will receive the 100 Euro. You can check the legal bases here. (link). The winners of the draw may donate the prize to one or more of the project(s) specified below or keep part or all of the 100 Euros

If you win the 100 Euros, your donation will be sent to the corresponding projects, and the Euros that you have not donated will be sent directly to you. If you are interested, we can send you the collective receipt of each of the donations.

If you win the 100 Euros, which amount will you donate to the following projects? (rotate NGO)

1. International project to address Covid-19: I donate _________Euro

2. International project to address climate change: I donate _________Euro

3. Spanish project to address Covid-19: I donate _________Euro

4. Spanish project to address climate change: I donate _________Euro

5. I keep _________Euro

Project 1 aims to generate better diagnostics, treatments and vaccines to reduce the expansion of Covid-19 and adapt to it internationally. [Click here in case you want more information.](https://www.who.int/es/emergencies/diseases/novel-coronavirus-2019/donate)

Project 2 aims to improve forecasts, infrastructure and energy technologies to reduce emissions and adapt to climate change internationally. [Click here in case you want more information](https://cutt.ly/IyEyCNL).

Project 3 aims to generate better diagnostics, treatments and vaccines to reduce the expansion of Covid-19 and adapt to it in Spain. [Click here in case you want more information](https://cutt.ly/ryEyJGB).

Project 4 aims to improve forecasts, infrastructure and energy technologies to reduce emissions and adapt to climate change in Spin. [Click here in case you want more information](https://cutt.ly/ryEyJGB).

How much do you believe the majority of the other collaborators have donated out of the 100 Euros in the previous questions?

1. International project to address Covid-19: I believe they donated _________Euro

2. International project to address climate change: I believe they donated _________Euro

3. Spanish project to address Covid-19: I believe they donated _________Euro

4. Spanish project to address climate change: I believe they donated _________Euro

5. I believe they kept _________Euro

Did you undertake any of the following actions during the confinement?

- Separate most of your waste for recycling
- Reduced energy consumption (e.g., less air conditioning or heating)
- Talked to others about environmental issues
- Avoid buying excessively packaged products
- Chosen a more environmentally friendly form of transport (walking, cycling, public transport, electric car)
- Purchased products with an environmental label
- Participating in a demonstration, attending a conference, partaking in an activity (e.g., the collective cleaning of a beach or park)
- Holiday trips by plane

How frequently have you undertaken these activities more or less during the confinement (compared to before the confinement)? (Less, about the same, more).

- Separate most of your waste for recycling
- Reduced energy consumption (eg lowering air conditioning or heating, leaving no appliances on stand-by, purchased energy efficient appliances)
- Talked to others about environmental issues
- Avoid buying excessively packaged products
- Chosen a more environmentally friendly form of transport (walking, cycling, public transport, electric car)
- Purchased products with an environmental label
- Participating in a demonstration, attending a conference, having been part of an activity (eg, the collective cleaning of a beach or park)
- Holiday trips by plane

How frequently would you like to undertake these activities more or less during the confinement (compared to before the confinement)? (Less, about the same, more).

- Separate most of your waste for recycling
- Reduced energy consumption (eg lowering air conditioning or heating, leaving no appliances on stand-by, purchased energy efficient appliances)
- Talked to others about environmental issues
- Avoid buying excessively packaged products
- Chosen a more environmentally friendly form of transport (walking, cycling, public transport, electric car)
- Purchased products with an environmental label
- Participating in a demonstration, attending a conference, having been part of an activity (eg, the collective cleaning of a beach or park)
- Holiday trips by plane

Are you concerned are you about climate change? (Response options: Not at all, a little, quite some, much, very much)

How much of a threat do you think climate change is to you and your family? (Response options: Not at all, a little, quite some, much, very much)

Overall, how would you rate your personal experience with climate change? (Response options: Very negative, negative, neutral, positive, very positve)

Under the Paris Agreement from 2015, each country, including Spain, must implement policies to reduce their CO_2_ emissions, which contribute to climate change. One major proposal to achieve emissions reduction is by implementing a carbon tax on fossil fuels whose combustion is the main cause of CO_2_ emissions.

¿How acceptable do you think a carbon tax is for reducing CO_2_ emissions? (Response options: completely unacceptable, somewhat unacceptable, neither unacceptable nor acceptable, somewhat acceptable, completely acceptable)

To the best of your knowledge, what percentage of the Spanish population would accept or not accept a carbon tax? Type three numbers from 0 (no one) to 100 (everyone). Please make sure that the total amount is equal to 100.

- % of people who accept the tax
- % of people who do not accept the tax
- % of people who are undecided

How acceptable do you find the following climate policies? (Response options: completely unacceptable, somewhat unacceptable, neither unacceptable nor acceptable, somewhat acceptable, completely acceptable)

- limits on carbon emissions by households based on their cars
- stricter energy efficiency standards for diesel and gasoline cars
- subsidies to buy electric cars
- banning gasoline and diesel cars in 2030
- educational programs for citizens about efficient use of gasoline and diesel cars

Carbon taxes generate revenues which can be used for different purposes. How acceptable do you find the carbon tax if its revenues were…? (Response options: completely unacceptable, somewhat unacceptable, neither unacceptable nor acceptable, somewhat acceptable, completely acceptable)

- used to compensate low-income households
- to support the development of climate projects
- returned in equal amount to all households as compensation
- used to pay for the extra public expenditures resulting from the Covid-19 crisis?

What percentage of the total carbon tax revenues (100%) would you prefer to allocate for each of the 4 proposed options? Please make sure that the total amount is equal to 100%.

- Support the development of climate projects
- Spend the revenues on extra public expenditures resulting from the Covid-19 crisis
- Return the revenues in equal amount to all households as compensation
- Return the revenues to compensate low-income households

How serious of a threat do you think Covid-19 is to you and your family? (Response options: not at all, little, considerable, much, very much)

Have you had serious health issues due to Covid-19? (Response options: yes, no)

How many of your family members and friends have had serious health issues due to Covid-19 (excluding yourself)

Did you undertake paid work during the Covid-19 confinement (specifically during April)? (Response options: No, I did not work, yes, I worked less than usual, yes, I worked as usual, yes, I worked more than usual)

Did you work from home during the confinement? (Response options: yes, no , partially

How would you rate your work productivity during the confinement compared to before the confinement? (Response options: much less productive, less productive, the same, more productive, much more productive)

To which extent have the Covid-19 pandemic and the confinement caused any psychological stress on you? (Response options: none, little, quite some, much, very much)

How did the Covid-19 crisis affect the net monthly income (including salary and/or rents) of your household? (Response options: very negatively, negatively, no effect, positively, very positively)

To which extent do you agree with the following statement? (Response options: Totally agree, partially agree, neutral, partially disagree, totally disagree)

- “Climate change has contributed to the Covid-19 outbreak” (Cursiva)

Overall, how do you evaluate your personal experience of Covid-19 confinement? (Response options: very negative, negative, neutral, positive, very positive)

Overall, how would you rate the performance of the government to face the Covid-19? (Response options: Very negative, negative, neutral, positive, very positive)

Overall, how would you rate the collaboration of the Spanish citizens to face the Covid-19 crisis? (Response options: Very negative, negative, neutral, positive, very positive)

Control question 2

A list of statements on GDP, growth and other related issues is now presented. All statements refer to rich, industrialized countries (European Union, United States, Canada etc.) unless otherwise stated. Could you please indicate your opinion on each statement? (Response options: strongly disagree, disagree, slightly disagree, not agree or disagree, slightly agree, agreed, strongly agree)

- Maintaining economic growth is essential to improving people's life satisfaction.
- Economic growth is necessary to finance environmental protection.
- Without economic growth the economy will be less stable.
- Economic growth is necessary to finance the public health system and the pension system.
- In view of limited natural resources, rich countries may have to forego economic growth to ensure that all poor people in the world can achieve a decent standard of living.

What is your current job situation? (Response options: I am currently working in a paid job, worker affected by a temporary suspension of employment (Spanish ERTE), student, unemployed, disability pension, retired, unpaid work (household maintenance, care work…)

What was your net monthly income of your household in the last year? (Response options: €1000 or less, between €1001-€2000, between €2001-€3000, between €3001-€4000, more than €4001, I prefer not to answer)

(show to 50% of sample with header, and the other 50% without the header)

A recent public opinion survey in Spain demonstrated that 43% of them would accept a carbon tax, 38% would not accept it, and 19% is undecided.

How acceptable do you find a carbon tax? (Response options: completely unacceptable, somewhat unacceptable, neither unacceptable nor acceptable, somewhat acceptable, completely acceptable)

Here are four positions on the relation between economic growth and the environment. Which of them comes closest to your own opinion? Public policy should ...

- … further pursue economic growth in spite of its environmental impacts.
- … further pursue economic growth. There are many ways to make economic growth compatible with environmental sustainability.
- … ignore economic growth as a policy aim, that is, be completely neutral about growth. This will amplify the policy spectrum to combine well-being and environmental sustainability goals.
- … stop pursuing economic growth. Production and consumption need to be reduced in an equitable way to achieve environmental sustainability.

Which values are important to you as principles guiding your life and which are less important? Try to distinguish as much as possible between the values using all the numbers. (Response options: -1=the value is the opposite of the principles that guide me, 0=the value is not important at all, it is not relevant as a guiding principle for me, 1=the value is very unimportant, 3=the value is important, 6= the value is very important, 7= value is of supreme importance as a guiding principle in my life)

- Universalism (openness, social justice, a peaceful world, equality)
- Benevolence (willingness to help, honesty, forgiveness, loyalty, responsibility)
- Respect for the Earth (respect rights of other species, be in harmony with other species)
- Environmental protection (minimizing pollution and waste, careful use of natural resources)

How trustworthy do you think politicians are? (Response options: very untrustworthy, somewhat untrustworthy, neutral, somewhat trustworthy, very trustworthy)

Where would you situate yourself ideologically? Use a scale ranging from 1 to 10, where 1 is ‘left-wing’ and 10 is ‘right-wing’ (Response options: 1=left wing, 2, 3, 4, 5, 6, 7, 8, 9, 10=right wing, don’t know/ I prefer not to answer)

¿Do you consider yourself more Spanish or more of a different nationality? Use a scale from 1 to 10, being 1 if you consider yourself totally Spanish, and 10 if you consider yourself totally from other nationality (Response options: 1=Spanish, 2, 3, 4, 5, 6, 7, 8, 9, 10=Other nationality, don’t know/ I prefer not to answer)


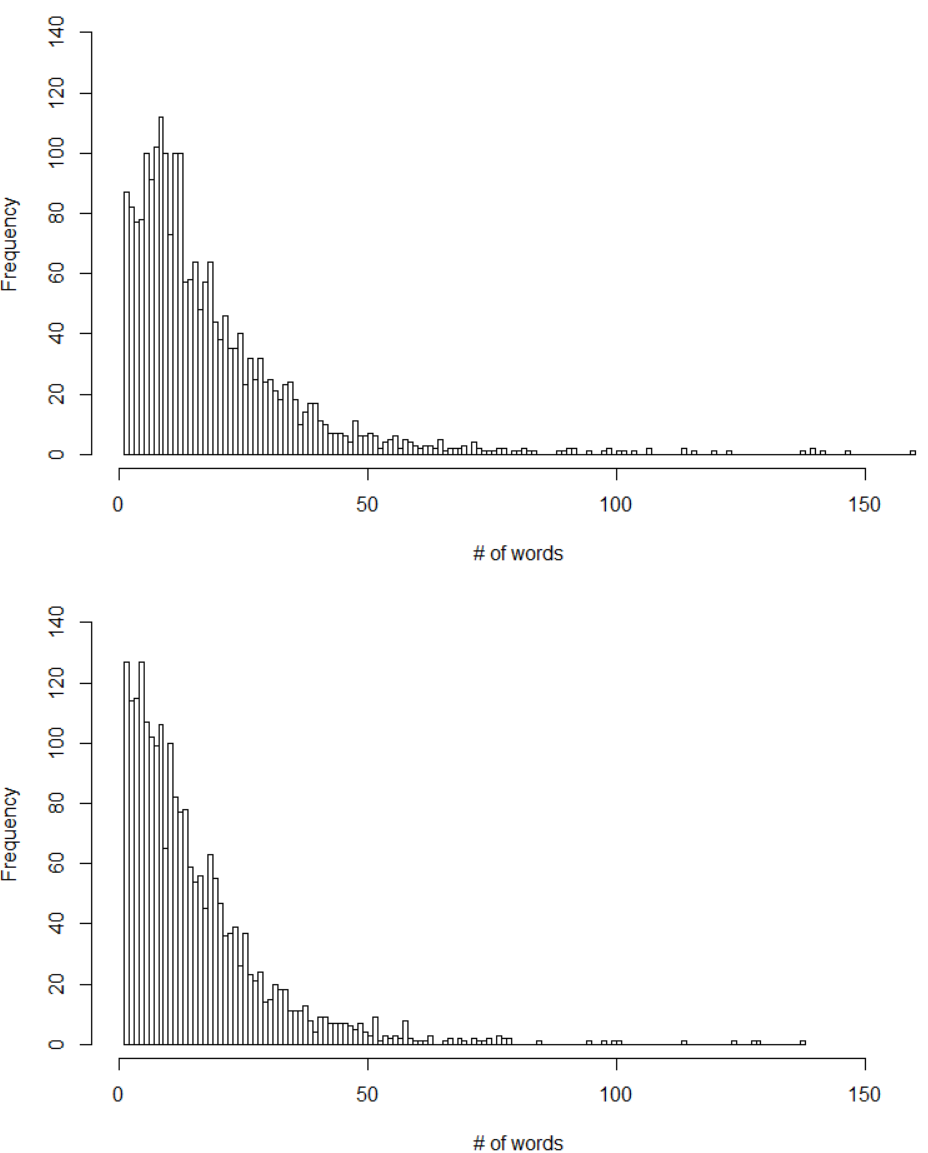


**S2 Fig. Length of responses to the two open-ended questions (first question in the upper plot and second in the lower one)**

Note: on the X-axis the shortest length of response is 1.

**S1 Table. Covariates used in the regression analysis**

| **Name of the variable** | **Exact wording of the question in the survey** |
| --- | --- |
| Gender | Information automatically provided by the survey company |
| Age | Information automatically provided by the survey company |
| Education | What is your education? |
| Expectations about governmental climate action | Two issues that receive a lot of attention nowadays are climate change and the Covid-19 (Coronavirus) pandemic. Overall, how do you think the Covid-19 pandemic will affect actions of the government towards climate change? |
| Expectations about people’s climate action | Overall, how do you think the Covid-19 pandemic will affect actions of the Spanish citizens towards climate change? (Response options: Very negatively, negatively, neutrally, positively, very positively) |
| Perceived threat from climate change | How much of a threat do you think climate change is to you and your family? |
| Perceived threat from COVID-19 | How serious of a threat do you think Covid-19 is to you and your family |
| Income change due to confinement | How did the Covid-19 crisis affect the net monthly income (including salary and/or rents) of your household? |
| Overall experience with COVID-19 | Overall, how do you evaluate your personal experience of Covid-19 confinement? |
| Evaluation of government fighting COVID-19 | Overall, how would you rate the performance of the government to face the Covid-19? |
| Evaluation of citizens fighting COVID-19 | Overall, how would you rate the collaboration of the Spanish citizens to face the Covid-19 crisis? |
| Carbon tax acceptance | How acceptable do you find a carbon tax? |


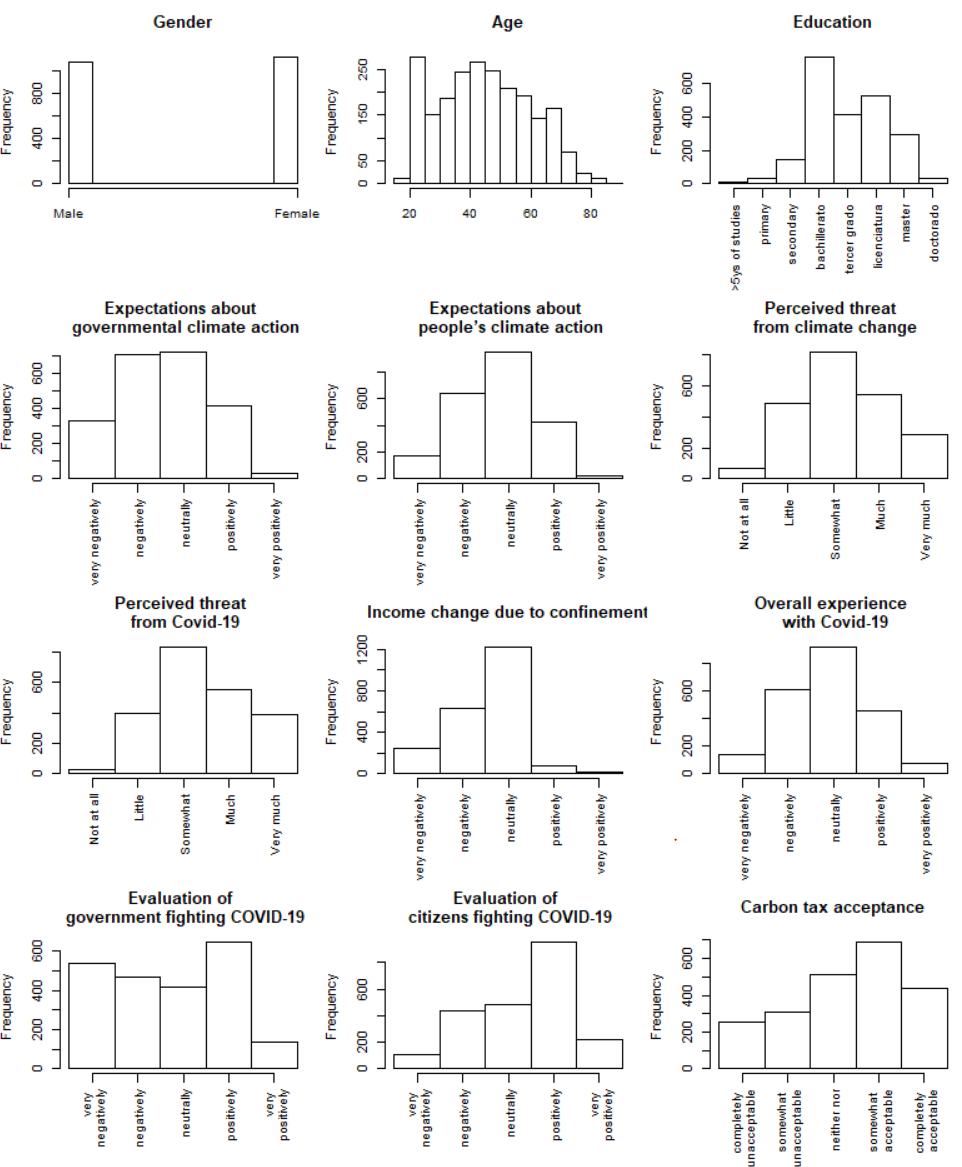


**S3 Fig. Distribution of values of covariates for the open-ended questions**

Note: for education “med prof form” means medium professional formation or university entrance level while “sup professional” means superior professional formation.

**S2 Table. Descriptive statistics on sociodemographic variables.**

| **Variables** | **Description** | **Mean (SD) or %** | **Spanish population** |
| --- | --- | --- | --- |
| Gender | Dummy: female | 51.0% | 50.99% |
| Age | 18 to 88 years old | 45.50 (15.09) | 43.59 |
| Monthly household income (last year) | 1 (less than 1000€) to 5 (More than 4000€) | 2.58 (1.14) translating into a value between  ± €2600 | 2385 euros per month |
| Education | 1 (Less than 5 years of school) to 8 (University) | 5.02 (1.32); 91.5% of the sample have medium professional or higher studies | 63.8% have a medium professional or higher studies (CIS, 2020)* |
| Political orientation | 1 (left-wing) to 10 (right-wing) | 4.44 (2.41) | 4.6 (2.0) (CIS, 2020)* |

Notes: Survey sampling was done by Netquest using quotas on age, gender and geographical distribution, making the sample representative for the general public in Spain on those characteristics. The remaining variables are compared with census data from the Spanish National Institute of Statistics ([www.ine.es](http://www.ine.es)) unless other source is indicated. We find that the sample is also representative for the other covariates, except for education and income.

* Source: Centro de Investigaciones Sociológicas (CIS), Barómetro de Junio 2020.


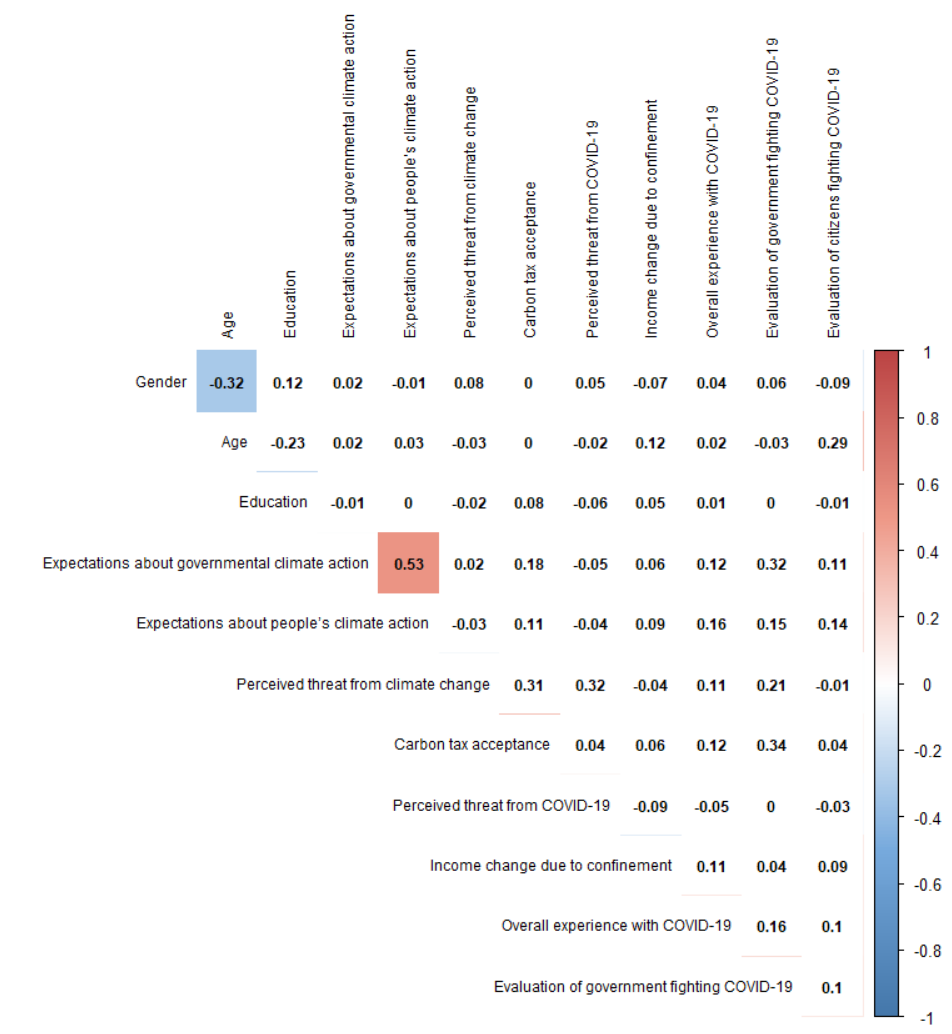


**S4 Fig. Testing pairwise correlations among covariates in our STM models.**

Note: Colors indicate correlations significant at 5% or better.


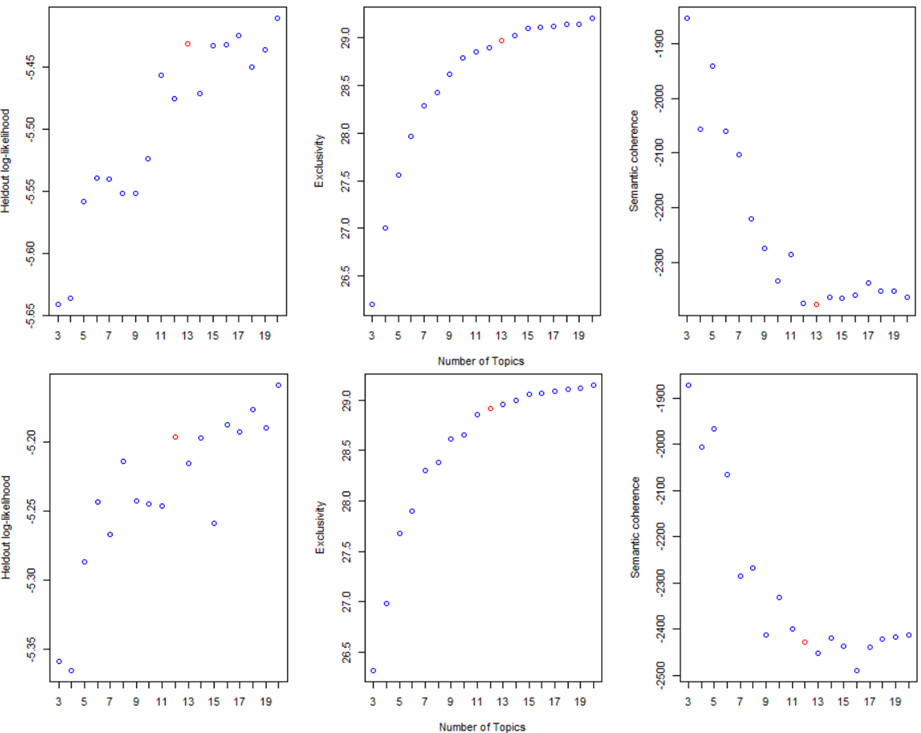


**S5 Fig. Model performance for distinct number of topics for the first (top panel) and the second (lower panel) open-ended questions**

**S3 Table. Topics identified for responses to the first open-ended on governmental climate action in Spanish**

|  | **Topic label** | **Most discriminating terms and illustrative responses** | **Topic proportion** |
| --- | --- | --- | --- |
| 1 | Insufficient resources for climate | *recurso, dinero, crisis, invertir, inversión, falta, dedicar, suficiente, requerir, económico* | 8.0% |
|  |  | *“No habrá recursos suficientes debido al gran coste económico de la crisis”* |  |
| 2 | COVID-19 and climate change are independent | *creer, afectar, seguir, cambiar, igual, diferente, aunque, cosa, influir, modificar* | 8.2% |
|  |  | *“En realidad no creo que una cosa afecte a la otra”* |  |
| 3 | Priority for COVID-19 | *atención, segundo_plano, relacionado, accion, cambio_climático, dejar, prestar, problema, covid, prioritario* | 12.5% |
|  |  | *“Porque van a dejar atrás todas las acciones respecto al cambio climático para centrarse en el covid”* |  |
| 4 | Government responds inadequately to COVID-19 | *hacer, relación, español, asunto, ninguno, capaz, desastre, gestionar, respecto, correctamente* | 5.3% |
|  |  | *“Simplemente porque el gobierno español no tiene ni la menor idea de actuar correctamente en estos casos así que españa se va a ir a una crisis muy severa”* |  |
| 5 | COVID-19 distracts from climate | *ahora, virus, pandemia, importancia, actualmente, nunca, importante, aprovechar, considerar, primero* | 5.6% |
|  |  | *“No creo que se tenga en cuenta el cambio climático mientras haya otra distracción sobre la mesa nunca le han dado la importancia que merece y obviamente con una pandemia en curso le dan menos todavía”* |  |
| 6 | Self-interested politicians | *saber, situación, pensar, pasar, preocupar, venir, gestión, importar, mismo, solo* | 6.0% |
|  |  | *“Porque a los políticos solo les interesa su bolsillo no están haciendo nada por nada ni por nadie excepto por ellos mismos”* |  |
| 7 | COVID-19 adds to waste | *más, mascarilla, plástico, guante, residuo, poner, material, desechable, tirar, mirar* | 8.1% |
|  |  | *”Uso de guantes y mascarillas son desechables y se generan muchos residuos”* |  |
| 8 | Teleworking and less travel | *medio_ambiente, transporte, mayor, contaminante, consumo, negativo, vehículo, energia, fomentar, privado* | 6.5% |
|  |  | *“Se incentivará el uso de transportes alternativos como la bicicleta y el teletrabajo de forma que se reducirá el número de desplazamientos a los puestos laborales”* |  |
| 9 | Priority for economy and health | *economía, política, prioridad, país, tener, salud, medioambiental, claro, primar, reactivar* | 7.9% |
|  |  | *“El gobierno tendrá que valorar que el empleo la economía y la salud son prioridades ante el cambio climático”* |  |
| 10 | Spend climate money on COVID-19 | *lucha, gasto, combatir, esfuerzo, priorizar, fondo, centrar, destinado, destinar, partida* | 5.1% |
|  |  | *”Porque parte del esfuerzo y medios económicos destinados a la lucha contra el cambio climático se destinarán a la lucha contra el covid”* |  |
| 11 | Government makes bad decisions | *político, mal, poco, decisión, vez, totalmente, mayoría, mucho, beneficio, único* | 8.2% |
|  |  | *“Porque en el gobierno hasta ahora no se han preocupado en colocar a las personas mejor preparadas para resolver los problemas que en españa vayan surgiendo solo se han preocupado en colocar a sus amistades”* |  |
| 12 | People stay at home and pollute less | *tomar, medida, contaminacioón, mantener, conciencia, esperar, adoptar, ver, disminuir, coche* | 8.9% |
|  |  | *”Gracias al covid y la cuarentena la gente no salía de casa no andaban en coche bajando así la contaminación”* |  |
| 13 | COVID-19 is environmental wake-up call | *confinamiento, cuenta, planeta, naturaleza, positivo, actividad, humano, volver, paron, haber* | 9.7% |
|  |  | *”Han notado la mejoría del planeta durante el confinamiento”* |  |

*Note:* The terms shown are those that are the most frequent as well as exclusive to each topic. Illustrative responses are chosen from the ten responses with the highest topic prevalence.

**S4 Table. Topics identified for responses to the second open-ended question on people’s climate action in Spanish**

|  | **Topic label** | **Most discriminating terms and illustrative responses** | **Topic proportion** |
| --- | --- | --- | --- |
| 1 | More awareness and less consumption | *tomar, salir, consumir, conciencia, contaminación, desplazar, aun, casa, consciente,* *necesitar* | 6.4% |
|  |  | *“La gente cada vez toma más conciencia de ello se usa mucho más la bicicleta se consume con más conciencia”* |  |
| 2 | People act as before | *hacer, seguir, mismo, poco, influir, ciudadano, tema, explicar, accion, covid* | 9.8% |
|  |  | *“Van a seguir actuando igual que lo hacían hasta el covid”* |  |
| 3 | Priority for economy and health | *cambiar, afectar, economia, relación, situación, salud, hábito, medioambiente, económico, cosa* | 7.1% |
|  |  | *”Que priorizamos salud o economía si decidimos por la salud no generamos dinero si priorizamos economía el virus se expande a quién le importa el cambio climático”* |  |
| 4 | Lack of government support | *gobierno, deber, ayudar, poner, medio, medioambiental, ciudadanía, ambiental, motivo, paro* | 6.1% |
|  |  | *“Porque la gente no está lo suficientemente concienciada del perjuicio que estamos ocasionando y deben ser los gobiernos los que obliguen con enseñanzas a la gente a actuar”* |  |
| 5 | Confinement fosters environmental care | *saber, cuidar, mejorar, mundo, dar_cuenta, aire, haber, humano, ciudad, grande* | 9.8% |
|  |  | *“La pandemia nos ha enseñado la importancia de respetar y cuidar el entorno y lo vulnerable que somos”* |  |
| 6 | Change is inevitable | *cambio, querer, tiempo, mucho, llevar, nuevo, virus, aspecto, sufrir, actividad* | 5.1% |
|  |  | *“La gente ha pasado tiempo en casa tiempo para reflexionar tiempo en el que darnos cuenta de que lo importante no lo podemos comprar con dinero y no todo vale creo que aprenderemos mucho de esta pandemia”* |  |
| 7 | Old habits die hard | *pensar, vida, cuanto, normalidad, costumbre, razon, sentido, creer, siempre, actitud* | 7.4% |
|  |  | *“Creo que a la larga se olvidarán y volverán a sus rutinas”* |  |
| 8 | People are myopic | *ahora, igual, importante, importar, bien, preocupación, atención, pendiente, recurso, proteger* | 7.8% |
|  |  | *“La gente vive día a día y les da igual lo que pase mañana mientras en el ahora estén bien”* |  |
| 9 | COVID-19 adds to waste | *mascarilla, guante, plástico, uso, tirar, residuo, suelo, generar, calle, público* | 13.1% |
|  |  | *“El uso masivo de mascarillas y guantes acabaran como basura en nuestros océanos”* |  |
| 10 | People are occupied by other problems | *cambio_climático, problema, preocupar, mayoría, preocupado, español, prioridad, existir, puesto, población* | 12.0% |
|  |  | *“Porque es otro problema y ya tenemos demasiados problemas”* |  |
| 11 | Teleworking and less travel | *menos, coche, respeto, desplazamiento, coger, cuenta, disminuir, viaje, ver, energía* | 9.2% |
|  |  | *“Se reducirá el transporte en coche y moto y se irá más en bicicleta y patinete se potenciará el teletrabajo evitando desplazamientos innecesarios”* |  |
| 12 | People consume more responsibly | *concienciado, naturaleza, aunque, concienciación, comportamiento, impacto, respuesta, poder, reflexionar, mejor* | 6.2% |
|  |  | *“Este confinamiento forzado a hecho que la gente interiorice que hay realizar una transición hacia un mundo más respetuoso con nuestro medio ambiente y con el exceso en el consumo”* |  |

Note: The terms shown are those that are the most frequent as well as exclusive to each topic. The original text was in Spanish. Illustrative responses are chosen from the ten responses with the highest topic prevalence.


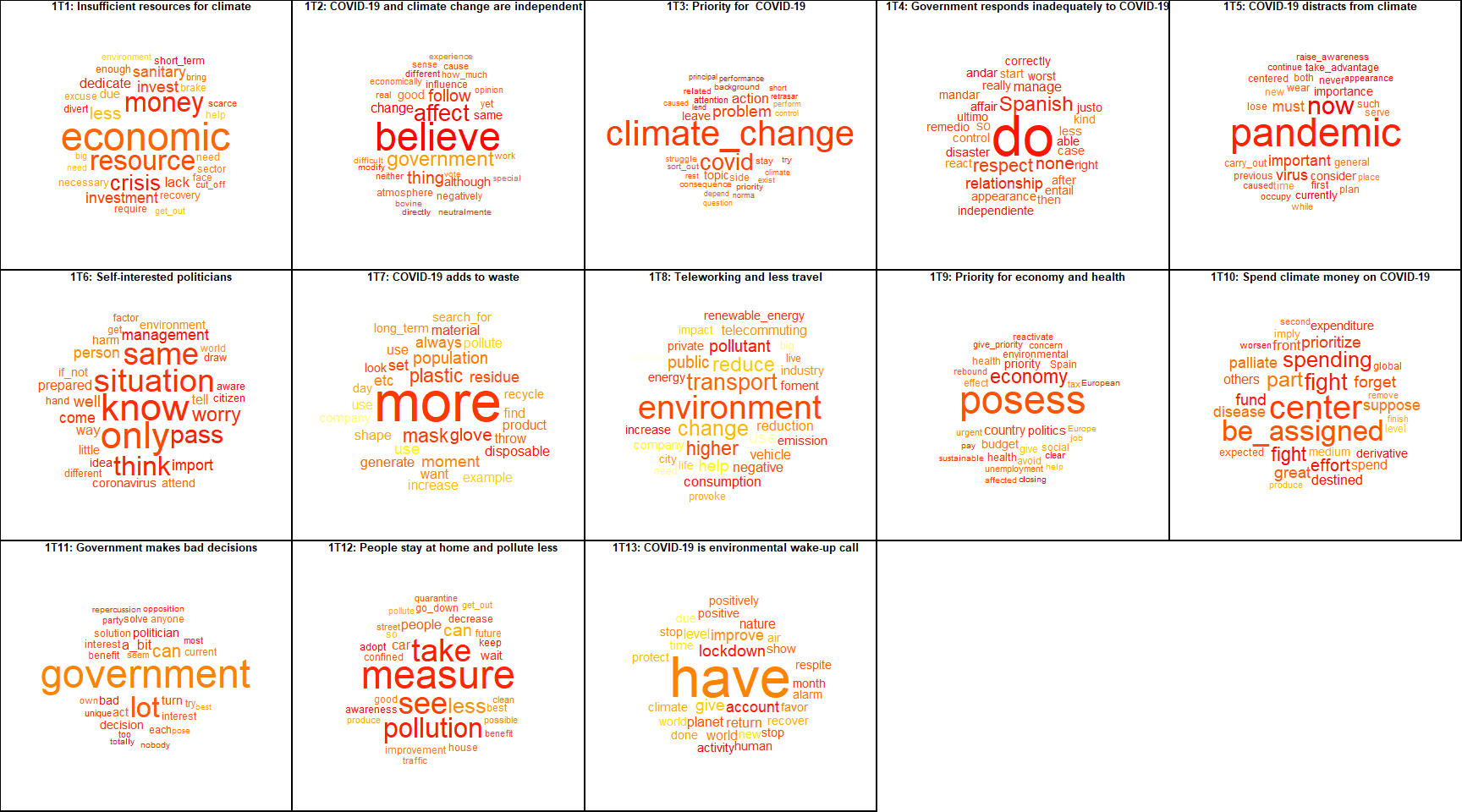


**S6 Fig. Word clouds of thirteen topics generated from the first open-ended question on governmental climate action**

Note: The font size reflects the probability (weight) of the respective word given the topic, while darker color of the word indicates its higher exclusivity.


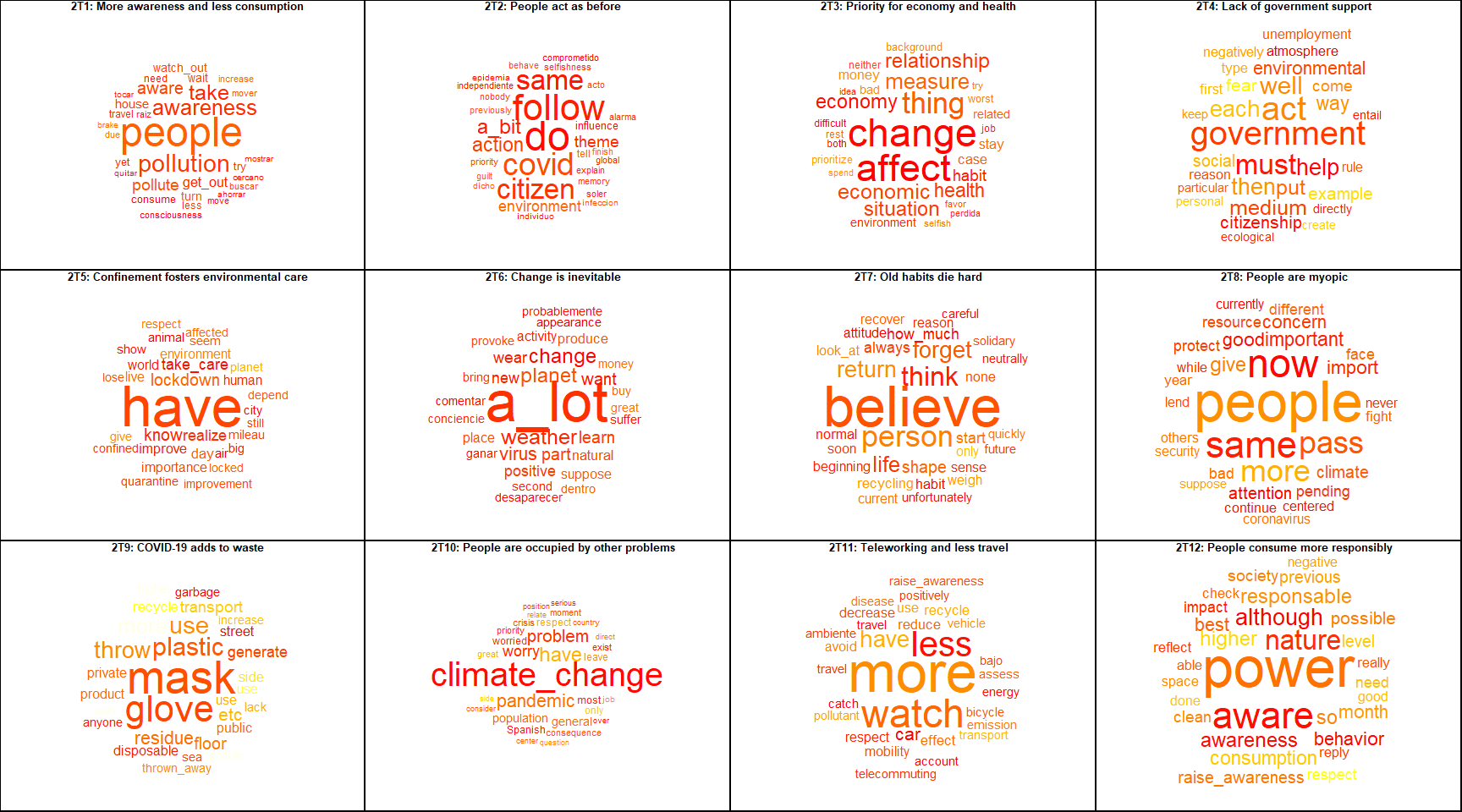


**S7 Fig. Word clouds of twelve topics generated from the second open-ended question on people’s climate action**

*Note:* The font size reflects the probability (weight) of the respective word given the topic, while darker color of the word indicates its higher exclusivity.


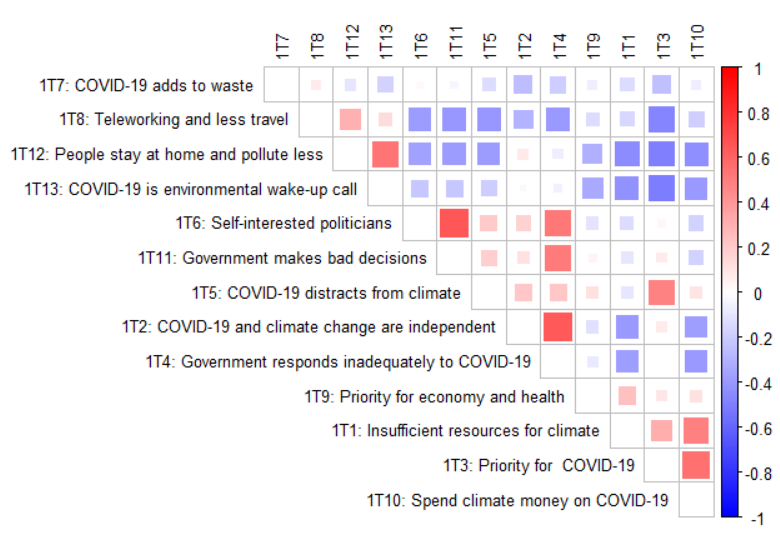


**S8 Fig. Co-occurrence of topics for the first open-ended question**

Note: Topics are ordered based on hierarchical clustering which positions stronger correlated topics closer.


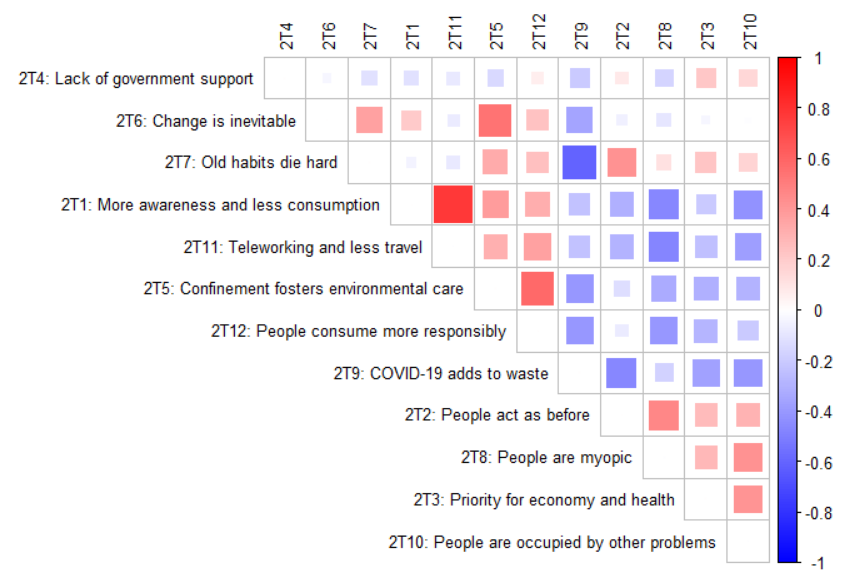


**S9 Fig. Co-occurrence of topics for the second open-ended question**

Note: Topics are ordered based on hierarchical clustering which positions stronger correlated topics closer.


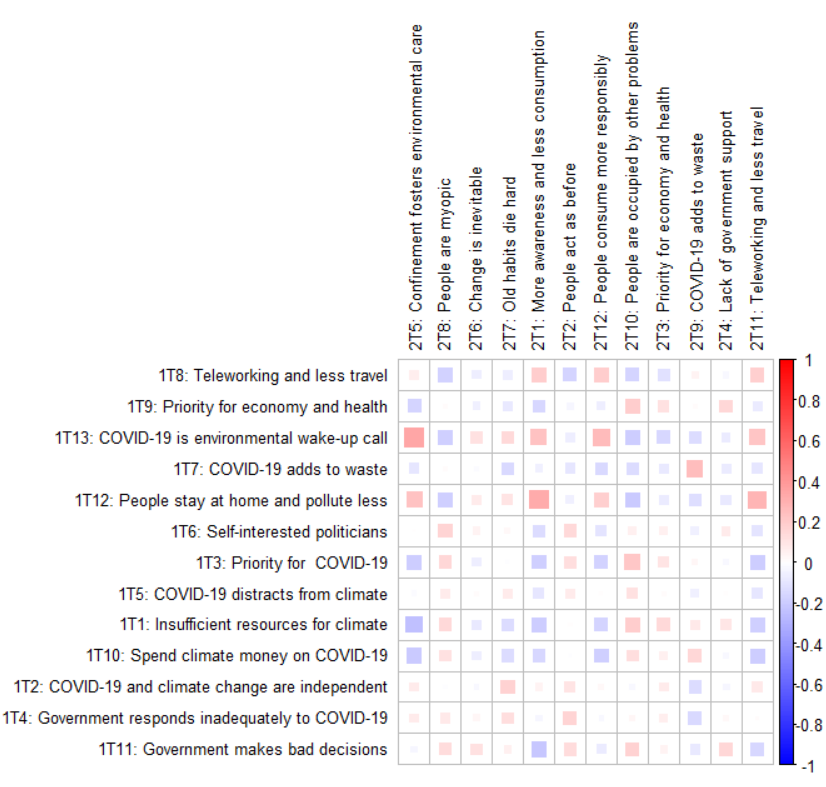


**S10 Fig. Co-occurrence of topics between the first and the second open-ended questions**

Note: the order of topics is adopted from S8-S9 Figs.

**S5 Table. Results of the regression analysis for the STM model based on the first open-ended question on governmental climate action**

|  | Intercept | Age | Gender | Education | Perceived threat from climate change | Perceived threat from COVID-19 | Overall experience with COVID-19 | Income change due confinement | Carbon tax acceptability | Expectations about governmental climate action | Evaluation of government fighting COVID-19 |
| --- | --- | --- | --- | --- | --- | --- | --- | --- | --- | --- | --- |
| 1T1 | 0.1478^***^ | 0.0002 | -0.0279^***^ | 0.0071^***^ | -0.0006 | 0.0028 | -0.0058^**^ | 0.0108^***^ | 0.0060^**^ | -0.0475^***^ | 0.0063^***^ |
| 1T2 | 0.0408^**^ | 0.0002 | 0.0156^***^ | -0.0027 | -0.0078^***^ | -0.0010 | 0.0035^*^ | -0.0013 | -0.0010 | 0.0189^***^ | -0.0018 |
| 1T3 | 0.1309^***^ | 0.0001 | 0.0087^*^ | 0.0021 | -0.0037 | -0.0022 | -0.0026 | 0.0070^**^ | 0.0052^***^ | -0.0235^***^ | 0.0043^**^ |
| 1T4 | 0.0871^***^ | 0.0002^*^ | 0.0041 | -0.0054^***^ | -0.0016 | -0.0006 | 0.0017 | 0.0012 | -0.0030^***^ | 0.0049^***^ | -0.0088^***^ |
| 1T5 | 0.0382^***^ | 0.0002^**^ | 0.0018 | -0.0009 | 0.0025^*^ | -0.0035^**^ | 0.0033^**^ | -0.0018 | 0.0001 | -0.0033^***^ | 0.0030^***^ |
| 1T6 | 0.1233^***^ | 0.0001 | 0.0021 | -0.0086^***^ | 0.0030 | -0.0055^*^ | 0.0001 | 0.0049^**^ | -0.0078^***^ | -0.0066^***^ | -0.0037^**^ |
| 1T7 | 0.1776^***^ | -0.0004^**^ | 0.0162^***^ | -0.0038^*^ | 0.0045^***^ | 0.0018 | -0.0114^***^ | -0.1021^***^ | -0.0027 | -0.0158^***^ | 0.0020 |
| 1T8 | 0.0510^*^ | -0.0007^***^ | -0.0255^***^ | 0.0151^***^ | 0.0032 | 0.0025 | 0.0083^***^ | -0.0038 | 0.0014 | 0.0299^***^ | 0.0008 |
| 1T9 | 0.0643^***^ | 0.0006^***^ | -0.0121^***^ | 0.0043^***^ | -0.0046^**^ | 0.0032 | 0.0010 | -0.0012 | 0.0086^***^ | -0.0153^***^ | -0.0004 |
| 1T10 | 0.0770^***^ | -0.0002 | 0.0037 | 0.0037^***^ | 0.0002 | 0.0016 | -0.0019 | -0.0007 | 0.0036^**^ | -0.0271^***^ | 0.0008^***^ |
| 1T11 | 0.1422^***^ | 0.0008^***^ | 0.0001 | -0.0051^***^ | 0.0063^***^ | -0.0045^***^ | -0.0039^**^ | -0.0008 | -0.0055^***^ | -0.0115^***^ | -0.0078^***^ |
| 1T12 | 0.0516^**^ | -0.0008^***^ | 0.0015 | -0.0031^*^ | -0.0029 | -0.0007 | -0.0020 | -0.0010 | -0.0024 | 0.0504^***^ | -0.0054^***^ |
| 1T13 | -0.0298 | -0.0002 | 0.0114^**^ | -0.0046^**^ | 0.0060^**^ | 0.0040 | -0.0016 | -0.0033 | -0.0024 | 0.0465^***^ | 0.0035 |

Note: Asterisks ^***^, ^**^, and ^*^ denote 1%, 5%, and 10% significance, respectively. Coefficients indicate whether prevalence of respective topics changes with the value of the covariates.

**S6 Table. Results of the regression analysis for the STM model based on the second open-ended question on people’s climate action**

|  | Intercept | Age | Gender | Education | Perceived threat from climate change | Perceived threat from COVID-19 | Overall experience with COVID-19 | Income change due confinement | Carbon tax acceptability | Expectations about people’s climate action | Evaluation of citizens fighting COVID-19 |
| --- | --- | --- | --- | --- | --- | --- | --- | --- | --- | --- | --- |
| 2T1 | 0.0037^***^ | -0.0009^***^ | -0.0024 | -0.0007 | 0.0040^***^ | 0.0005 | -0.0017 | -0.0067^***^ | 0.0015 | 0.0277^***^ | -0.0006 |
| 2T2 | 0.1983^***^ | -0.0003^**^ | -0.0127^***^ | -0.0083^***^ | 0.0022 | -0.0021 | -0.0014 | 0.0034 | -0.0080^***^ | 0.0052^**^ | -0.0065^***^ |
| 2T3 | 0.1455^***^ | 0.0001 | -0.0112^***^ | 0.0036^**^ | -0.0150^***^ | 0.0055^***^ | -0.0025 | -0.0036 | -0.0005 | -0.0097^***^ | 0.0004 |
| 2T4 | 0.0871^***^ | 0.0008^***^ | -0.0211^***^ | 0.0020 | -0.0004 | 0.0064^***^ | -0.0003 | 0.0050^*^ | 0.0001 | -0.0054^**^ | 0.0005 |
| 2T5 | 0.0151 | -0.0002 | 0.0149^***^ | -0.0073^***^ | 0.0059^**^ | -0.0005 | 0.0005 | -0.0070^**^ | -0.0003 | 0.0363^***^ | -0.0001 |
| 2T6 | 0.0752^***^ | -0.0001 | 0.0024 | -0.0022^***^ | 0.0051^***^ | -0.0037^***^ | -0.0055^***^ | -0.0014 | 0.0004 | 0.0017 | -0.0015 |
| 2T7 | 0.0349^**^ | 0.0003^**^ | 0.0095^***^ | -0.0009 | 0.0021 | - 0.0068^***^ | -0.0035^**^ | 0.0032 | -0.0018 | 0.0195^***^ | -0.0045^***^ |
| 2T8 | 0.2389^***^ | -0.0001 | -0.0024 | -0.0021 | -0.0075^***^ | -0.0027 | 0.0010 | 0.0021 | -0.0095^***^ | -0.0230^***^ | -0.0055^**^ |
| 2T9 | 0.3130^***^ | -0.0012^***^ | 0.0326^***^ | 0.0060 | 0.0025 | -0.0022 | 0.0158^***^ | -0.0007 | 0.0129^***^ | -0.1127^***^ | 0.0091^*^ |
| 2T10 | 0.0871^***^ | 0.0014^***^ | -0.0112^**^ | 0.0059^***^ | -0.0087^***^ | 0.0051^*^ | -0.0039 | 0.0075^*^ | 0.0036 | -0.0220^***^ | 0.0015 |
| 2T11 | -0.0934^***^ | -0.0001 | -0.0001 | -0.0018 | 0.0024 | 0.0044^*^ | 0.0037 | -0.0060^**^ | -0.0006 | 0.0525^***^ | 0.0056^**^ |
| 2T12 | -0.0819^***^ | 0.0004^***^ | 0.0019 | 0.0023 | 0.0074^***^ | -0.0040^**^ | -0.0021 | 0.0043^**^ | 0.0022 | 0.0299^***^ | 0.0017 |

Note: Asterisks ^***^, ^**^, and ^*^ denote 1%, 5%, and 10% significance, respectively. Coefficients indicate whether prevalence of respective topics changes with the value of the covariates.
