# Supplementary material for: Public expectations about the impact of COVID-19 on climate action by citizens and government
Source: PLoS One. 2022 Jun 9;17(6):e0266979. doi: 10.1371/journal.pone.0266979 (PMC9182260; doi:10.1371/journal.pone.0266979)
Supplement: Attachment — Submitted filename: Comments.pdf [file pone.0266979.s002.pdf]

The article addresses the critical issue of public expectations about the impact of COVID-19 on climate action by citizens and government. The article is interesting and is of high quality. However, I have some below-mentioned concerns.

1. In the section "2.1 Data collection" the authors stated that "In this study we draw on two open-ended questions from an online survey conducted in June-July 2020 in Spain." The author should explicitly mention what kind of online platforms were used to collect responses, e.g., social media (Facebook, LinkedIn, Twitter, WhatsApp, etc.) and/or email? Although the authors have mentioned, "The survey was conducted in Spanish language by a professional panel survey company "Netquest. " But I still believe the individual data collection sources should be indicated.
2. What techniques were employed to cope with the sample bias?
3. The authors used an online survey in this study, which is unquestionably a more convenient, robust, and cost-effective survey method. However, the authors should acknowledge in the paper that these types of surveys have limitations. For example, authors can mention the survey's inability to reach difficult populations such as the elderly and those with no educational background.
4. Furthermore, a discussion section should be included that interprets and describes the significance of your findings in light of what was already known about the research problem being investigated, as well as to explain any new understanding or fresh insights about the problem after you've taken the findings (any novel or unexpected results) into consideration. In my opinion, the inclusion of a discussion section can add to the quality of the findings of your article.
5. Finally, there are some typo errors that should be corrected in the revised version.
