# Supplementary material for: Public expectations about the impact of COVID-19 on climate action by citizens and government
Source: PLoS One. 2022 Jun 9;17(6):e0266979. doi: 10.1371/journal.pone.0266979 (PMC9182260; doi:10.1371/journal.pone.0266979)
Supplement: Attachment — Submitted filename: Comments.docx [file pone.0266979.s003.docx]

**Decision: Major Revision**

**Comments:**

In this paper “Public expectations about the impact of COVID-19 on climate action by citizens and government” authors presented the Spanish citizen expectation about the COVID-19 impact on climate action by the public and government. This paper is a good attempt at understanding public opinion regarding climate action and the pandemic impact on climate impact. There are several gaps that still need to be addressed in this paper.

Major question that the author needs to answer

1. Authors should explain why they have not included the United Nations - Sustainable Development Goals (SDGs). It would be better to know if the citizen has an understanding regarding UN 17 SDG goals and their opinion regarding COVID 19 impact on these goals.
2. Paper lacks a detailed discussion regarding their results. They just explain the methodology and conclude their findings. They should at least include a one-page discussion by linking their findings with other research publications.
3. Author should create the link with other related studies and explain what measurements should be taken by the government to change the negative impact of the public regarding the Impact of COVID 19. For Example, European Investment Bank conducted a survey and found 82% of Spanish people demand strict government regulation regarding climate change. (<https://www.eib.org/en/press/all/2021-360-81-percents-of-spanish-people-in-favour-of-stricter-government-measures-imposing-behavioural-changes-to-address-the-climate-emergency>). Authors should incorporate these surveys results and include some research findings in other countries in their discussion section.
4. Similarly, UNDP conducted the world’s largest survey (University of Oxford, 2021) regarding public opinion regarding climate change. Authors can relate their findings on how COVID 19 has impacted these survey results.
5. This paper lacks some flow charts and detailed discussion. Authors can study and refer to this paper by (Hoque et al., 2019) for what kind of adaptive measurements can be implemented by the government to reduce climate change impact. Referring to this paper as an example to write their discussion section.
6. There are several places where sentences are grammatically incorrect or not easy to understand. Some of the sentences are mentioned in the minor correction section.
7. Almost all figures’ captions or labels are not readable. It is advisable to correct the labels and captions. Sometimes captions are going inside the figure.

**Minor Corrections**

**Introduction**

**Page 2 – Paragraph 3 Line 4** – It is better to mention the year as well. It will be easy for the readers to understand which year authors are talking about.

**Page 2 – Paragraph 4 Line 9** – Please correct this sentence “the first aims to identify the”

**Materials and Methods**

**Data collection**

**Page 3 Paragraph 1 Line 5** – Please use full abbreviation whenever using it the first time. (i.e. SD M).

**Page 3 Paragraph 1 Line 9** - Please correct grammar “around 40 questions and is participants took on”

**Page 3 Paragraph 1 Line 7** – Writers are saying their subjects are highly educated and earn higher earnings. They should also mention how much percentage they are talking about. What about people from low income and less educated people. What about their ratio.

**Page 4 Paragraph 8 Line 2** – Please add “WORD” after (12) in this line “for the second 16 (12). In both questions”

**Data Analysis**

**Page 5 Paragraph 2 Line 3** - Please add “Total” between “14381 words” in this line “while the remaining responses contain 612 (unique) words and 14381 words”.

**Page 8 and Page 8 Table 1** – Incorrect Inverted commas at some places

**Page 9 Paragraph 2** – Please correct this paragraph grammatically - “Compared to the basic socio-demographic characteristics like age, gender and education, perceived threat from COVID-19 and climate change, as well as overall personal experience with COVID-19-related confinement and its effects on household income, do not explain much variation in topics people expressed in their open responses.”

**Page 10 – Figure 1** – It is suggested to write the X-axis label at the bottom instead of at each figure’s top.

**Page 11 – Section 3.1** – The title of this section is not making any sense according to the paragraph below. Kindly correct the title “3.2 Associations with people’s climate action”

**Page 13** – Please correct the grammar – “People who perceive climate as less of a threat than COVID-19 express more the opinion that people prioritize economy and health (2T3) and talk less about the need to care for the planet (2T6).”

**Page 16 – Figure 3** – label at the x-axis is not visible. Also please write full abbreviation instead of wrt in both y-axis label and x-axis. Legend circle indicating government and people should be easier to see. It should be bigger.
