# Supplementary material for: Public expectations about the impact of COVID-19 on climate action by citizens and government
Source: PLoS One. 2022 Jun 9;17(6):e0266979. doi: 10.1371/journal.pone.0266979 (PMC9182260; doi:10.1371/journal.pone.0266979)
Supplement: Attachment — Submitted filename: Response to Reviewers_final.docx [file pone.0266979.s004.docx]

# Responses to the Editor and Reviewers

# Title: "Public expectations about the impact of COVID-19 on climate action by citizens and government”

# Manuscript ID: PONE-D-22-02316 *PLOS ONE*

# We are grateful to the editor and the referees for their positive assessment of our paper and the valuable comments, which helped us to improve the paper. Below we explain how we have addressed each comment.

**Response to Journal Requirements:**

**1. 1. Please ensure that your manuscript meets PLOS ONE's style requirements, including those for file naming. The PLOS ONE style templates can be found at**

**https://journals.plos.org/plosone/s/file?id=wjVg/PLOSOne_formatting_sample_main_body.pdf and**

**https://journals.plos.org/plosone/s/file?id=ba62/PLOSOne_formatting_sample_title_authors_affiliations.pdf.**

We have checked the PLOS ONE style templates and adjusted our manuscript accordingly.

**2. Thank you for stating the following in the Acknowledgments Section of your manuscript:**

**[This work was funded by an ERC Advanced Grant from the European Research Council (ERC) under the European Union’s Horizon 2020 Research and Innovation Programme [grant agreement n° 741087] and by a María de Maeztu Program for Units of Excellence awarded to ICTA-UAB by the Spanish Ministry of Science and Innovation. I.S. acknowledges financial support from the Russian Science Foundation [RSF grant number 19-18-00262]. S.V.-T. acknowledges financial support from the Spanish Department of Science and Innovation [RYC-2017-22782].]**

**We note that you have provided funding information that is currently declared in your Funding Statement. However, funding information should not appear in the Acknowledgments section or other areas of your manuscript. We will only publish funding information present in the Funding Statement section of the online submission form.**

**Please remove any funding-related text from the manuscript and let us know how you would like to update your Funding Statement. Currently, your Funding Statement reads as follows:**

**[This work was funded by an ERC Advanced Grant from the European Research Council (ERC) under the European Union’s Horizon 2020 Research and Innovation Programme [grant agreement n° 741087] and by a María de Maeztu Program for Units of Excellence awarded to ICTA-UAB by the Spanish Ministry of Science and Innovation. I.S. acknowledges financial support from the Russian Science Foundation [RSF grant number 19-18-00262]. S.V.-T. acknowledges financial support from the Spanish Department of Science and Innovation [RYC-2017-22782]]**

**Please include your amended statements within your cover letter; we will change the online submission form on your behalf.**

Thank you, we updated our acknowledgement section and double-checked the funding statement.

**3. We note that you have stated that you will provide repository information for your data at acceptance. Should your manuscript be accepted for publication, we will hold it until you provide the relevant accession numbers or DOIs necessary to access your data. If you wish to make changes to your Data Availability statement, please describe these changes in your cover letter and we will update your Data Availability statement to reflect the information you provide.**

We have uploaded the relevant data and the code to reproduce the results at the following link at GitHub <https://github.com/IvanVSavin/PLOS_One_PublicExpectationsCOVID.git>

**4. While revising your submission, please upload your figure files to the Preflight Analysis and Conversion Engine (PACE) digital diagnostic tool,** [**https://pacev**](https://pacev)**2.apexcovantage.com/. PACE helps ensure that figures meet PLOS requirements. To use PACE, you must first register as a user. Registration is free. Then, login and navigate to the UPLOAD tab, where you will find detailed instructions on how to use the tool. If you encounter any issues or have any questions when using PACE, please email PLOS at figures@plos.org. Please note that Supporting Information files do not need this step.**

We have transformed our figures using PACE tool to the formats compatible with PLOS One requirements.

**Response to Reviewer 1:** (*our response is in italics*).

In this paper “Public expectations about the impact of COVID-19 on climate action by citizens and government” authors presented the Spanish citizen expectation about the COVID-19 impact on climate action by the public and government. This paper is a good attempt at understanding public opinion regarding climate action and the pandemic impact on climate impact.

*Thank you for your positive evaluation.*

There are several gaps that still need to be addressed in this paper.

Major question that the author needs to answer

1.	Authors should explain why they have not included the United Nations - Sustainable Development Goals (SDGs). It would be better to know if the citizen has an understanding regarding UN 17 SDG goals and their opinion regarding COVID 19 impact on these goals.

*We believe what you propose here is a new research question, different from our original goal implemented in the manuscript. As we explain in our paper, there are many studies discussing and speculating how one existential threat (COVID-19) will affect actions regarding another existential threat (namely, climate change). Our aim was to address this question by classifying and understanding public perception in this regard. While analyzing the effect of COVID-19 on SDGs may be an interesting question per se, this would be a completely different study than the one we prepared. Asking respondents about SDGs would fit better in a study that examines the relationship between COVID-19 and the broader aim of sustainable development.*

2.	Paper lacks a detailed discussion regarding their results. They just explain the methodology and conclude their findings. They should at least include a one-page discussion by linking their findings with other research publications.

*Thank you for this suggestion. We have added a new Discussion section (which is about two pages) where we interpret our findings and compare them with those of the existing literature.*

3.	Author should create the link with other related studies and explain what measurements should be taken by the government to change the negative impact of the public regarding the Impact of COVID 19. For Example, European Investment Bank conducted a survey and found 82% of Spanish people demand strict government regulation regarding climate change. (https://www.eib.org/en/press/all/2021-360-81-percents-of-spanish-people-in-favour-of-stricter-government-measures-imposing-behavioural-changes-to-address-the-climate-emergency). Authors should incorporate these surveys results and include some research findings in other countries in their discussion section.

*Thank you for this suggestion. We mention this survey’s results now in the Discussion section of our manuscript. We further stress here that due to increased public support for climate policy, governments can use the momentum and introduce stricter climate regulation.*

4.	Similarly, UNDP conducted the world’s largest survey (University of Oxford, 2021) regarding public opinion regarding climate change. Authors can relate their findings on how COVID 19 has impacted these survey results.

*Thank you, we now also compared our results to those of the UNDP survey.*

5.	This paper lacks some flow charts and detailed discussion. Authors can study and refer to this paper by (Hoque et al., 2019) for what kind of adaptive measurements can be implemented by the government to reduce climate change impact. Referring to this paper as an example to write their discussion section.

*We do not understand you suggestion well. The only flow chart we could find in the enclosed paper is in Figure 1 on socio-ecological framework of drinking water services, but this is quite far from our research focus. Also providing suggestions on climate policy mitigation and adaptation goes far beyond our paper’s scope. As we write in Conclusion, “**In summary, our study can be considered as a snapshot of public expectations, regarding future climate action by government and people affected by COVID-19, against which future developments can be compared.” In the new Discussion section, we try to provide some suggestions on policy implementation given increased climate policy support from many survey participants. We keep this modest, however, as we do not like to speculate about this topic too much.*

6.	There are several places where sentences are grammatically incorrect or not easy to understand. Some of the sentences are mentioned in the minor correction section.

*Thank you. We have carefully checked the manuscript once again. We corrected the phrases you indicated as well as a few other errors (mainly typos).*

7.	Almost all figures’ captions or labels are not readable. It is advisable to correct the labels and captions. Sometimes captions are going inside the figure.

*We have updated figures in our manuscript, in particular Figures 1-4 and S6-S7. However, font size in certain cases is necessarily small to fit the message on one figure. The possibility to enlarge figures both in the PDF format and online will allow readers to see the captions and labels in larger letter size.*

Minor Corrections

Introduction

Page 2 – Paragraph 3 Line 4 – It is better to mention the year as well. It will be easy for the readers to understand which year authors are talking about.

*We added the year.*

Page 2 – Paragraph 4 Line 9 – Please correct this sentence “the first aims to identify the”

*We corrected this to “the first aim to identify”.*

Materials and Methods

Data collection

Page 3 Paragraph 1 Line 5 – Please use full abbreviation whenever using it the first time. (i.e. SD M).

*We added an explanation that these indicate standard deviation and mean.*

Page 3 Paragraph 1 Line 9 - Please correct grammar “around 40 questions and is participants took on”

*We reformulated to “The survey contained about 40 questions and participants took on average 19 minutes to complete it.”*

Page 3 Paragraph 1 Line 7 – Writers are saying their subjects are highly educated and earn higher earnings. They should also mention how much percentage they are talking about. What about people from low income and less educated people. What about their ratio.

*We provide this information in S2 Table in the Supporting information, which was already referred to in the main text. It is true that in online surveys less educated people (and those with lower income) tend to be underrepresented. This is why we mention this limitation in our text more explicitly now.*

Page 4 Paragraph 8 Line 2 – Please add “WORD” after (12) in this line “for the second 16 (12). In both questions”

*Added.*

Data Analysis

Page 5 Paragraph 2 Line 3 - Please add “Total” between “14381 words” in this line “while the remaining responses contain 612 (unique) words and 14381 words”.

*Added. Thank you for pointing this out.*

Page 8 and Page 8 Table 1 – Incorrect Inverted commas at some places

*Corrected.*

Page 9 Paragraph 2 – Please correct this paragraph grammatically - “Compared to the basic socio-demographic characteristics like age, gender and education, perceived threat from COVID-19 and climate change, as well as overall personal experience with COVID-19-related confinement and its effects on household income, do not explain much variation in topics people expressed in their open responses.”

*Corrected.*

Page 10 – Figure 1 – It is suggested to write the X-axis label at the bottom instead of at each figure’s top.

*We moved the labels to the bottom.*

Page 11 – Section 3.1 – The title of this section is not making any sense according to the paragraph below. Kindly correct the title “3.2 Associations with people’s climate action”

*The title of the section is correct, but we adapted formulation of the first sentence in the paragraph to “**We now repeat the described steps of analysis for the responses to the second open question concerning which associations respondents have with future climate actions by people after having experienced the COVID-19 pandemic.”*

*Also, we adapted the names of the two sections to “Respondents’ associations…”*

Page 13 – Please correct the grammar – “People who perceive climate as less of a threat than COVID-19 express more the opinion that people prioritize economy and health (2T3) and talk less about the need to care for the planet (2T6).”

*Corrected.*

Page 16 – Figure 3 – label at the x-axis is not visible. Also please write full abbreviation instead of wrt in both y-axis label and x-axis. Legend circle indicating government and people should be easier to see. It should be bigger.

*Labels have been increased, abbreviations replaced, legends made clearer.*

**Response to Reviewer 2:** (*our response is in italics*).

The article addresses the critical issue of public expectations about the impact of COVID-19 on climate action by citizens and government. The article is interesting and is of high quality.

*Thank you for your positive evaluation.*

However, I have some below-mentioned concerns.

1. In the section "2.1 Data collection" the authors stated that "In this study we draw on two open-ended questions from an online survey conducted in June-July 2020 in Spain." The author should explicitly mention what kind of online platforms were used to collect responses, e.g., social media (Facebook, LinkedIn, Twitter, WhatsApp, etc.) and/or email? Although the authors have mentioned, "The survey was conducted in Spanish language by a professional panel company "Netquest." But I still believe the individual data collection sources should be indicated.

*Thanks, we have now added: “**The survey company Netquest hosts a large panel of about 1.3 million people living in 23 countries. Data collection was conducted in accordance with the general Market Research ISO norm 25252 using own software developments for online surveys (*[*https://www.netquest.com/en/online-surveys-investigation*](https://www.netquest.com/en/online-surveys-investigation)*)”. The survey respondents participate through a special software developed by the company and not by email or an online social network. We added this information in the section on data collection.*

2. What techniques were employed to cope with the sample bias?

*We did not use any additional techniques to deal with sample bias on level of education. As we earlier stated in the manuscript, “the sample includes 2200 participants and is representative of the general population in terms of age, gender and geographical distribution”. Making our sample representative in terms of other characteristics was not our aim.*

3. The authors used an online survey in this study, which is unquestionably a more convenient, robust, and cost-effective survey method. However, the authors should acknowledge in the paper that these types of surveys have limitations. For example, authors can mention the survey's inability to reach difficult populations such as the elderly and those with no educational background.

*. As we show in S2 Table in Supporting information, our sample covers population between 18 and 88 years old being representative of the general population of Spain in terms of age. However, you are right that people with no or little education are underrepresented in our analysis. We add a not on this in Section on Data collection.*

4. Furthermore, a discussion section should be included that interprets and describes the significance of your findings in light of what was already known about the research problem being investigated, as well as to explain any new understanding or fresh insights about the problem after you've taken the findings (any novel or unexpected results) into consideration. In my opinion, the inclusion of a discussion section can add to the quality of the findings of your article.

*We have added a Discussion section to the paper where we discuss our findings and embed them within the broader literature.*

5. Finally, there are some typo errors that should be corrected in the revised version.

*Thank you. We have carefully checked the manuscript once again.*
